# Supplementary figures and images for: HMGB1-BoxA gene therapy in reversing cisplatin resistance in non-small cell lung cancer
Source: PLoS One. 2025 Jun 25;20(6):e0327144. doi: 10.1371/journal.pone.0327144 (PMC12193834; doi:10.1371/journal.pone.0327144)

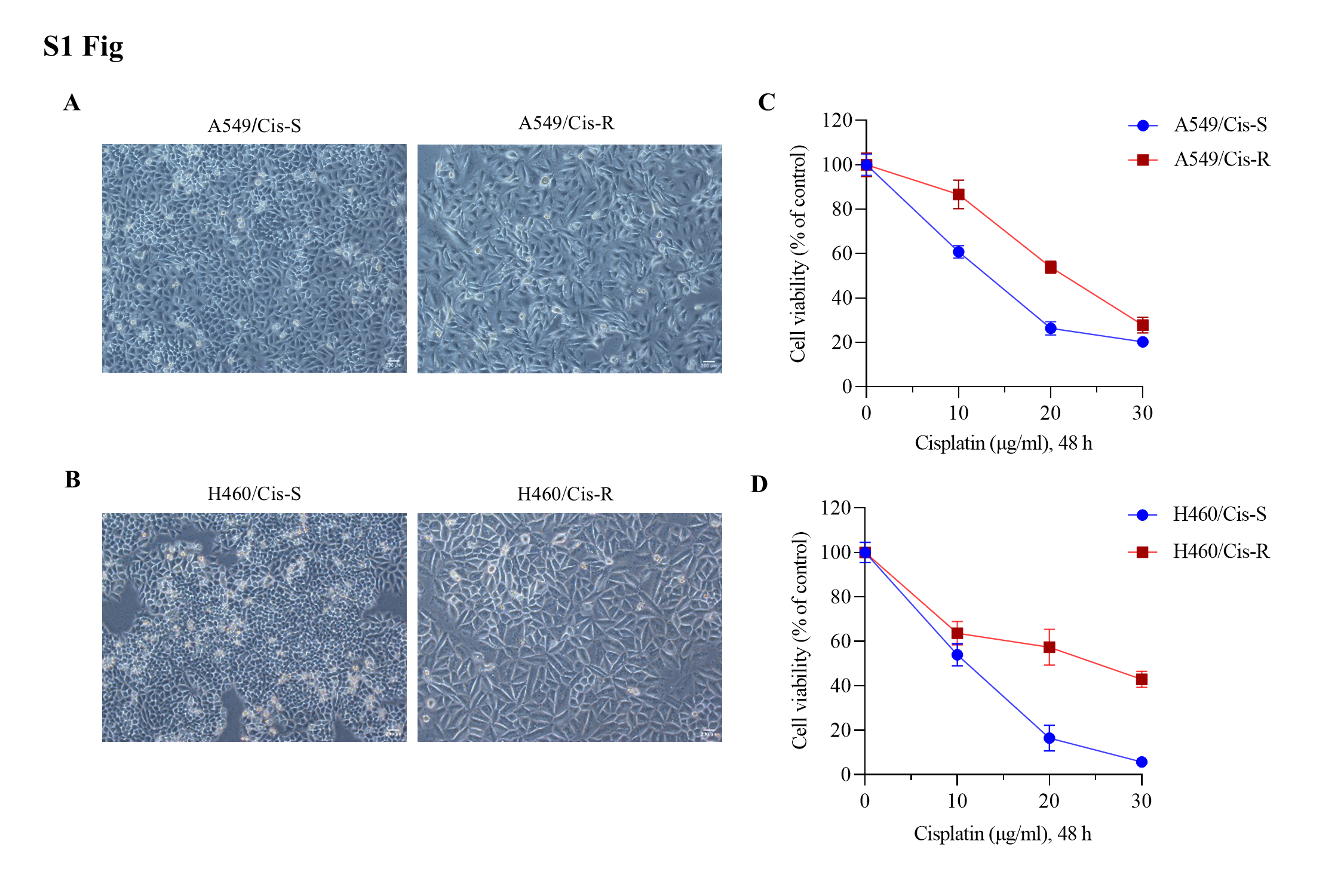

Supplement: S1 Fig — Representative image of A549/Cis-R (A), H460/Cis-R (B), and their Cis-S cells. Cisplatin sensitivity of A549/Cis-R and A549/Cis-S cells (C), H460/Cis-R and H460/Cis-S cells (D). Data are presented as mean ± SD of three independent experiments. (TIF) [file pone.0327144.s001.tif]

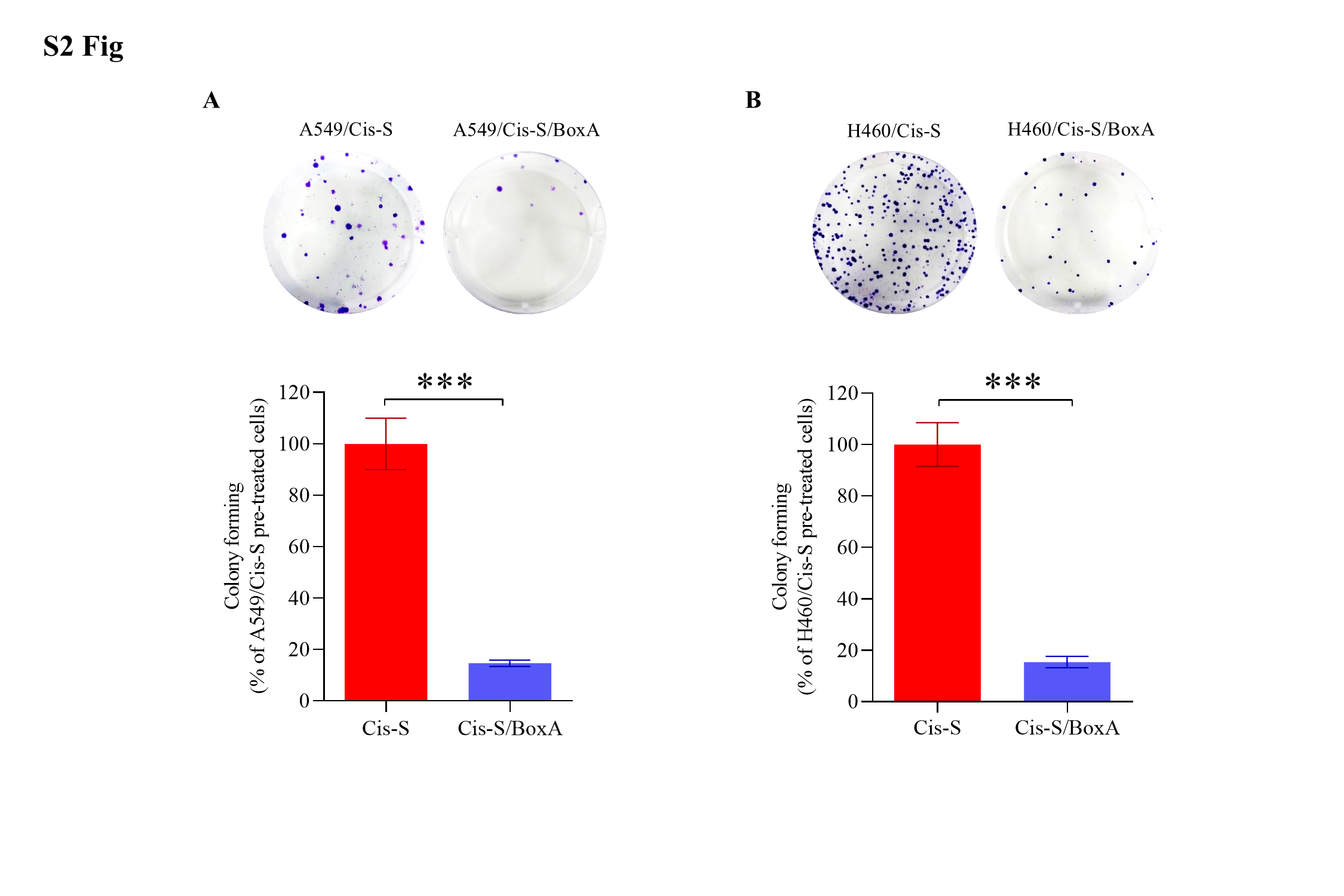

Supplement: S2 Fig — Representative images and colony-forming efficiency of A549/Cis-S/BoxA (A), and H460/Cis-S/BoxA (B) pretreated with 5 µg/ml cisplatin compared to the control-pretreated cells. Bar charts present mean ± SD. Statistically significant is indicated by *** P < 0.001. (TIF) [file pone.0327144.s002.tif]

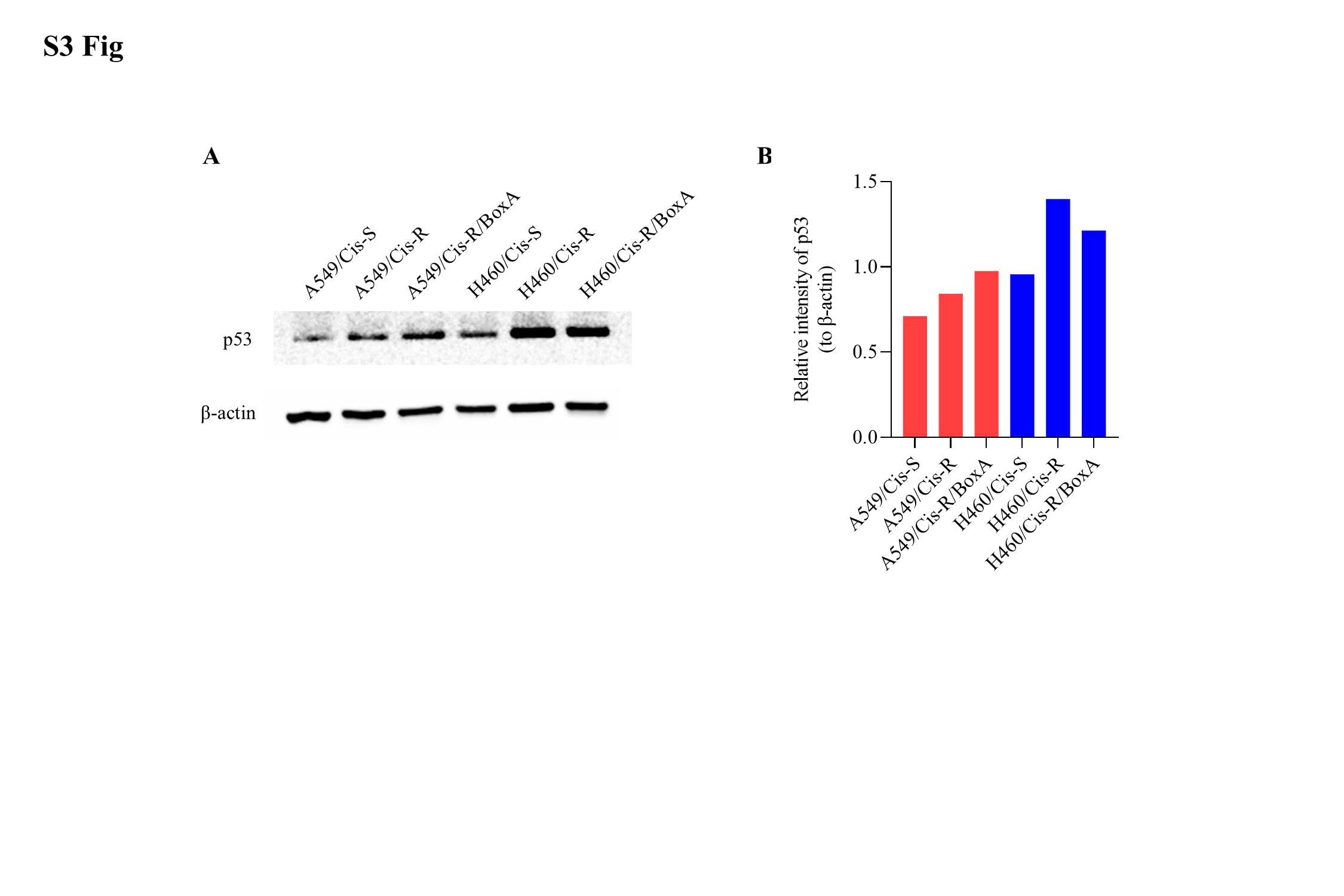

Supplement: S3 Fig — Protein expression levels of p53 in A549/Cis-S, A549/Cis-R, A549/Cis-R/BoxA, H460/Cis-S, H460/Cis-R, and H460/Cis-R/BoxA cells were evaluated by western blot (A). Relative protein intensity of p53 to protein loading controls β-actin in indicated samples (B). (TIF) [file pone.0327144.s003.tif]

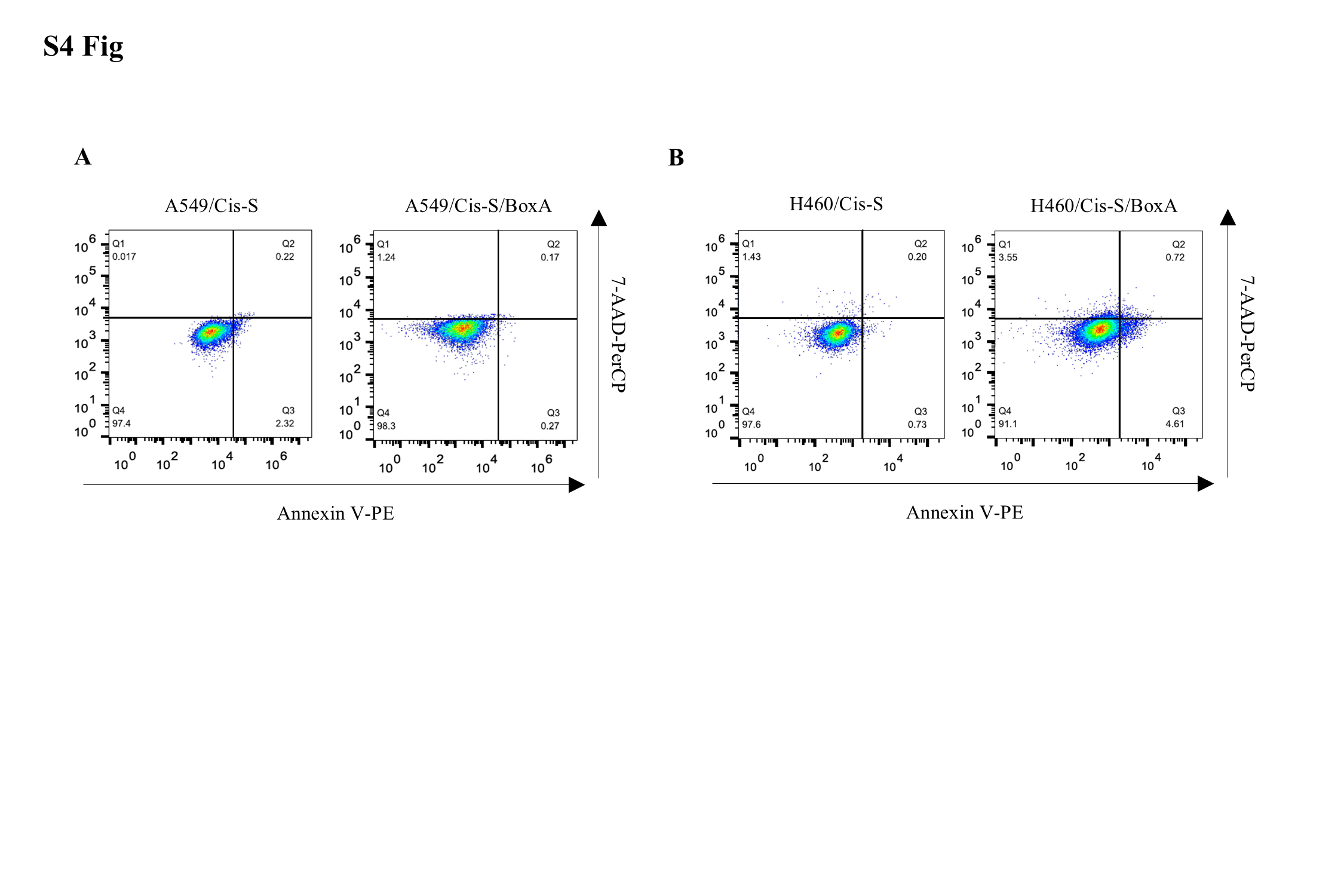

Supplement: S4 Fig — A549/Cis-S/BoxA (A), H460/Cis-S/BoxA (B), and their control cells were determined by apoptosis by using Annexin V/7-AAD staining and flow cytometry analysis. Flow cytometry histograms show the percentage rate of necrosis (Q1: 7-AAD + /AnnexinV−), late apoptosis (Q2: 7-AAD + /AnnexinV+), early apoptosis (Q3: 7AAD − /AnnexinV+) and live cells (Q4: 7-AAD − /AnnexinV−). (TIF) [file pone.0327144.s004.tif]

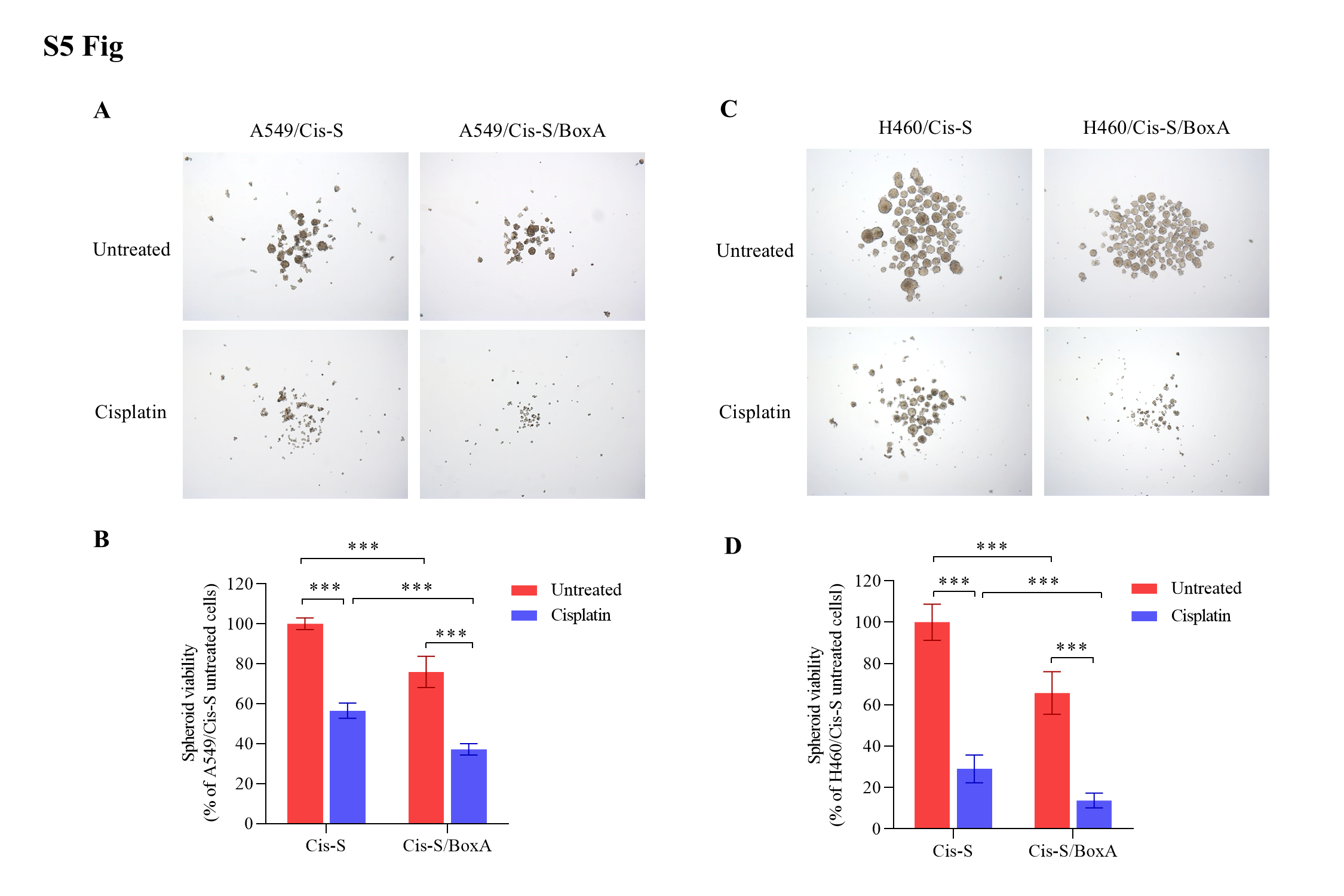

Supplement: S5 Fig — Representative spheroids images and relative spheroid viability of BoxA-existing cells, A549/Cis-S/BoxA (A, B), H460/Cis-S/BoxA (C, D) and their control cells with or without pretreated 5 ug/ml of cisplatin, Bar charts present mean ± SD. Statistically significant is indicated by *** P < 0.001. (TIF) [file pone.0327144.s005.tif]
