# Supplementary material for: HMGB1-BoxA gene therapy in reversing cisplatin resistance in non-small cell lung cancer
Source: PLoS One. 2025 Jun 25;20(6):e0327144. doi: 10.1371/journal.pone.0327144 (PMC12193834; doi:10.1371/journal.pone.0327144)

Supporting Information; The original images

HMGB1-BoxA gene therapy in reversing cisplatin resistance in non-small cell lung cancer

Nattapong Puthdee[1], Van-Hieu Mai[2, 3], Sirapat Settayanon[4, 5], Pithi Chanvorachote[6], Chatchawit Aporntewan[7], Apiwat Mutirangura[5], Chanida Vinayanuwattikun[8*]

[1]C2F-Postdoctoral Fellowship, Chulalongkorn University, Bangkok, Thailand.

[2]Faculty of Medicine, University of Health Sciences, Vietnam National University Ho Chi Minh City, Vietnam.

[3]Graduate Program in Clinical Sciences, Faculty of Medicine, Chulalongkorn University, Bangkok, Thailand.

[4]Interdisciplinary Program of Biomedical Sciences, Graduate School, Chulalongkorn University, Bangkok, Thailand.

[5]Center of Excellence in Molecular Genetics of Cancer and Human Diseases, Department of Anatomy, Faculty of Medicine, Chulalongkorn University, Bangkok, Thailand.

[6]Cell-Based Drug and Health Product Development Research Unit and Department of Pharmacology and Physiology, Faculty of Pharmaceutical Sciences, Chulalongkorn University, Bangkok, Thailand.

[7]Department of Mathematics and Computer Sciences & Omics Sciences and Bioinformatics Center, Faculty of Science, Chulalongkorn University, Bangkok, Thailand.

[8]Division of Medical Oncology, Department of Medicine, Faculty of Medicine, Chulalongkorn University and The King Chulalongkorn Memorial Hospital, Bangkok, Thailand.

*Corresponding Author, Email: Chanida.vi@chula.ac.th (CV)

The original blot image for S3 Fig.

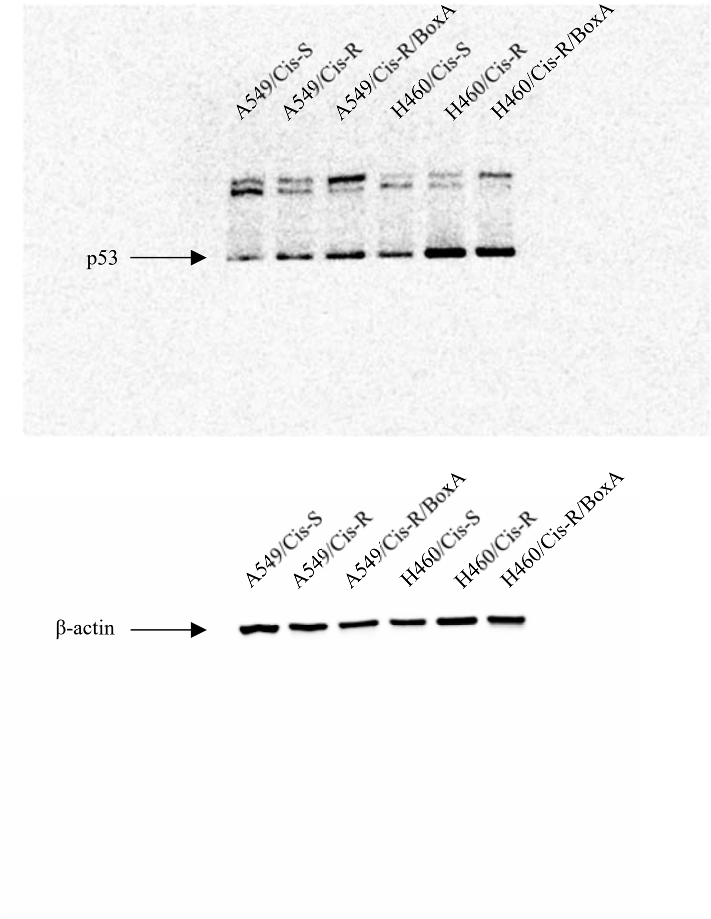

The original blot image for Fig 4A.

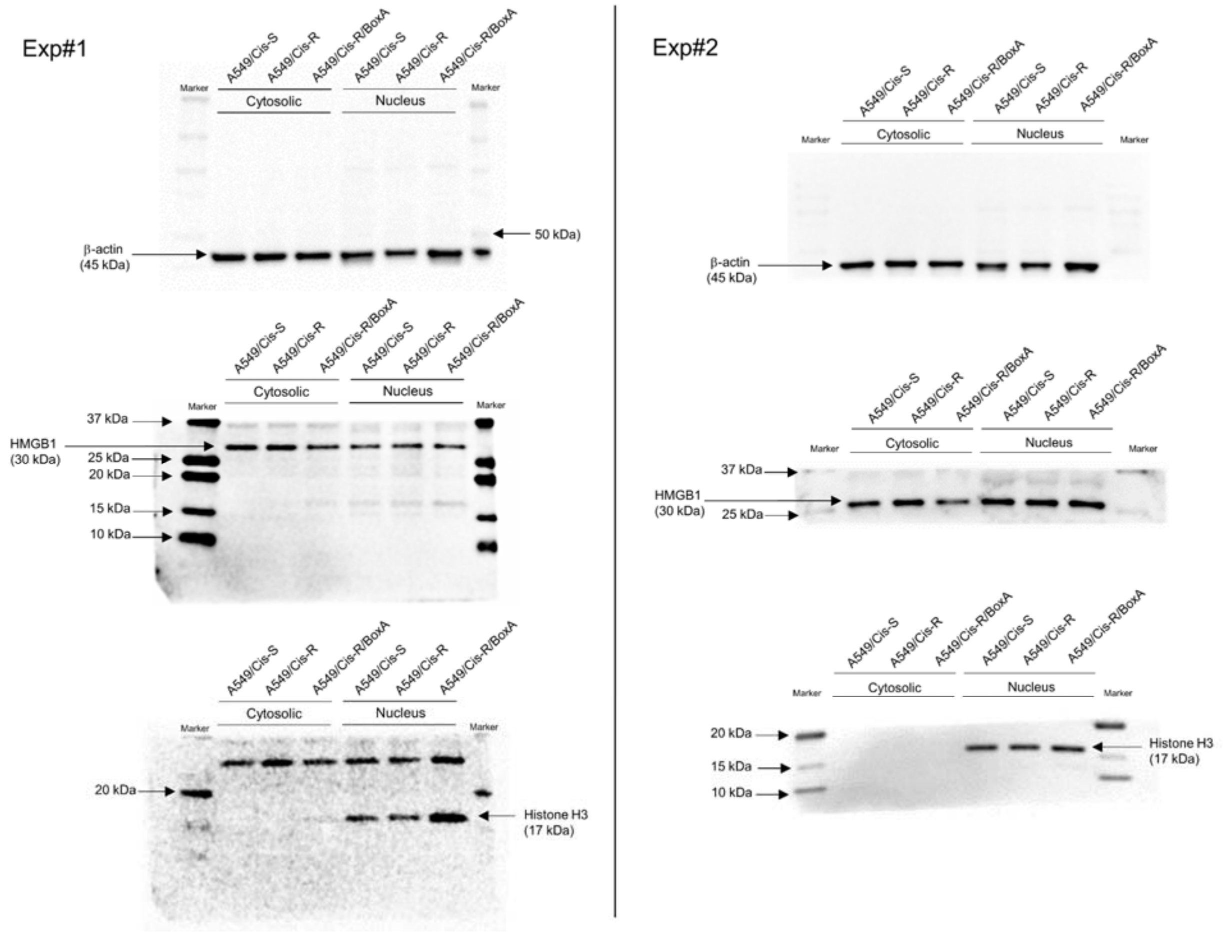

The original blot image for Fig 4C.

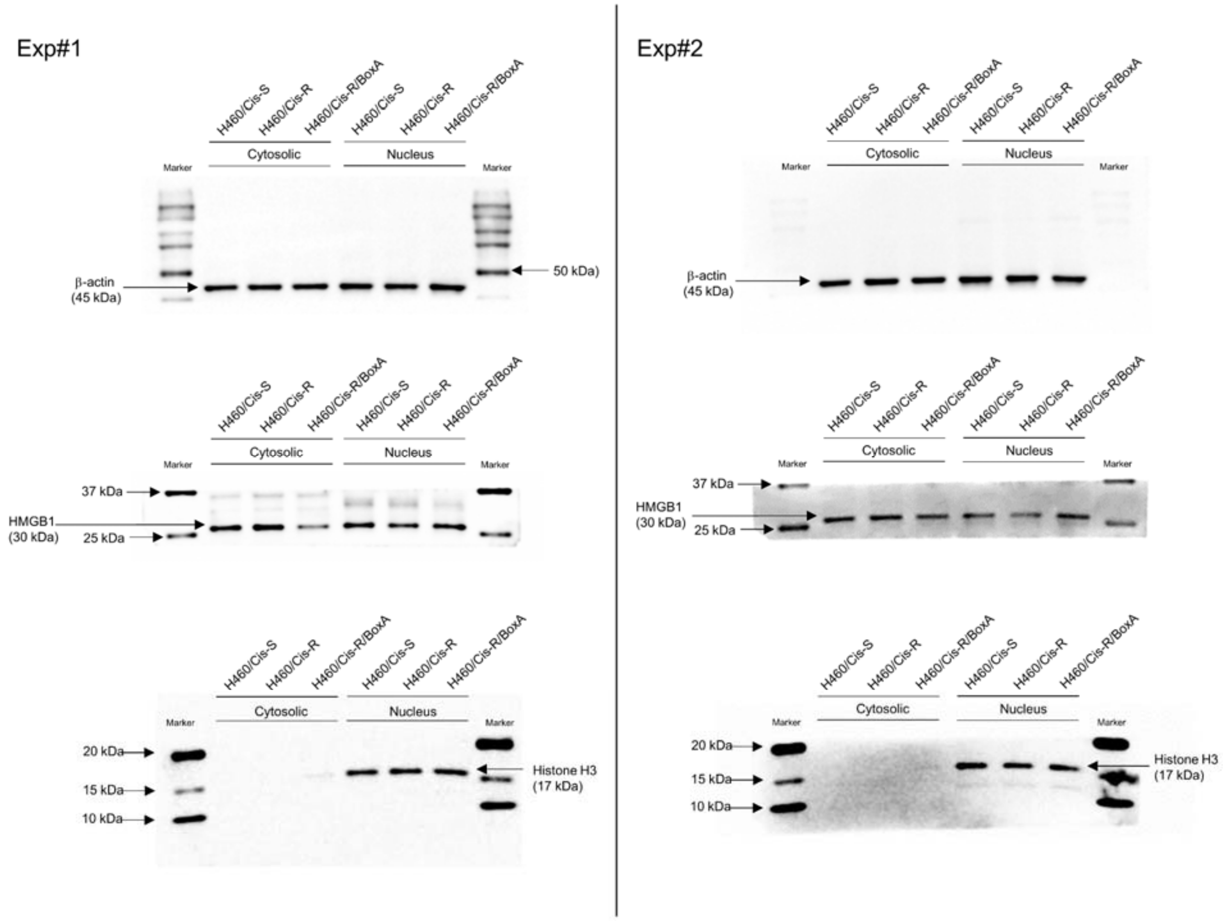

Supporting information: Representative image of colony formation

Fig 1C

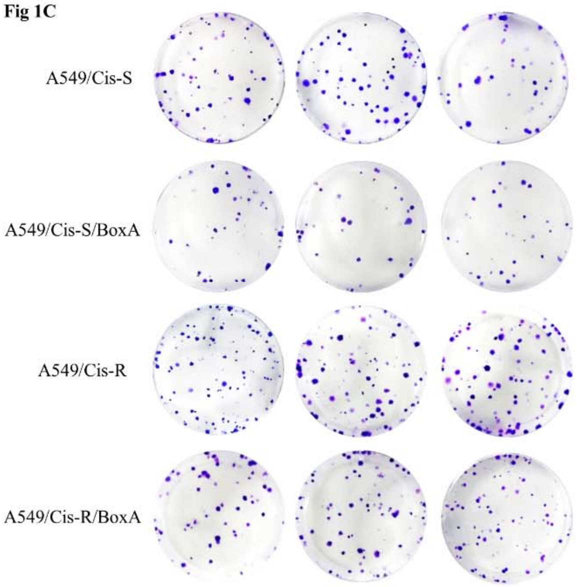

Fig 1E

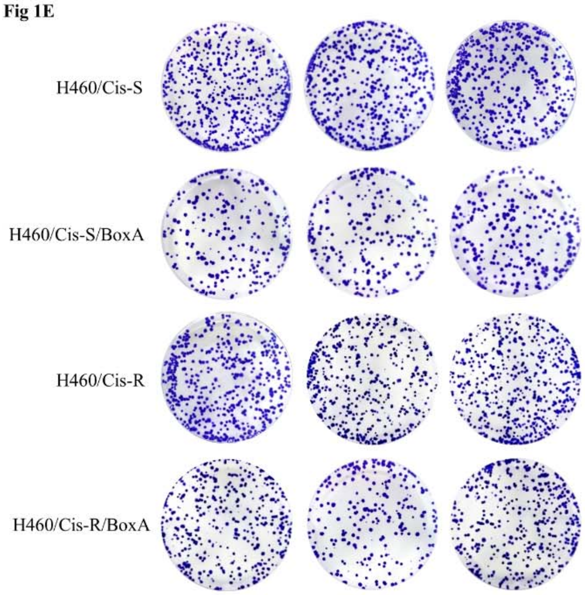

Supporting information: Representative image of colony formation

Fig 2C

A549/Cis-R_ untreated

A549/Cis-R_ cisplatin

A549/Cis-R/BoxA_ untreated

A549/Cis-R/BoxA_ cisplatin

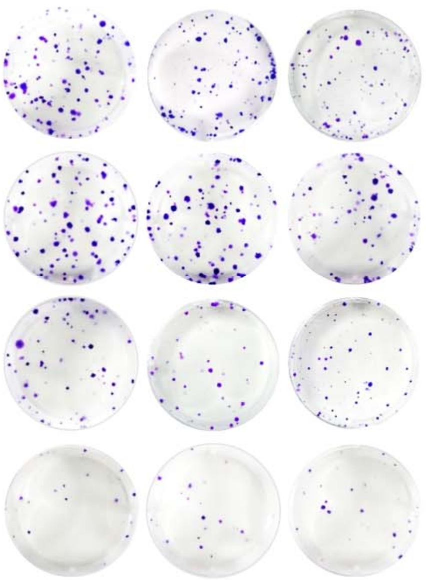

Fig 2E

H460/Cis-R_ untreated

H460/Cis-R_ cisplatin

H460/Cis-R/BoxA_ untreated

H460/Cis-R/BoxA_ cisplatin

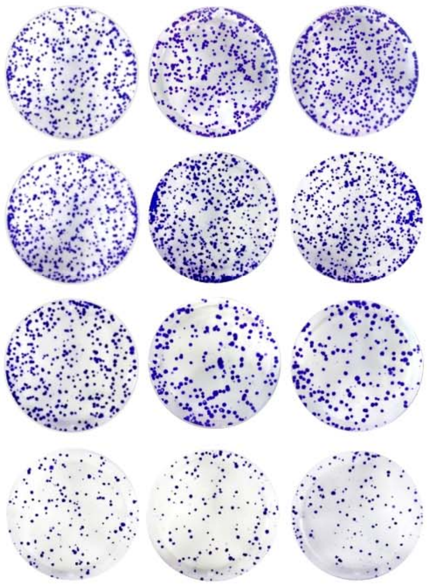

Supporting information: Representative image of colony formation

S2A_Fig

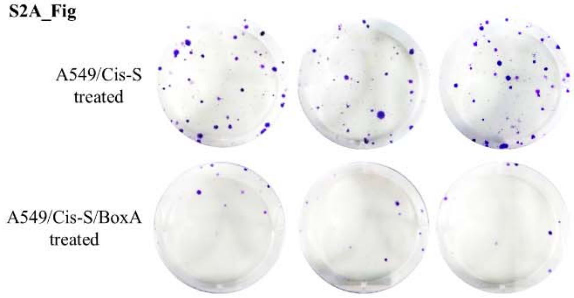

S2B_Fig

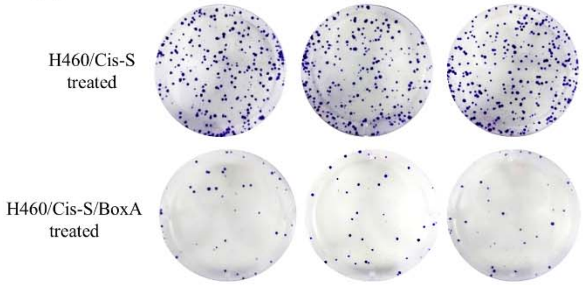

Supporting information: Representative image of sphere formation corresponding to Fig 3A

A549/Cis-R_
untreated

A549/Cis-R_
cisplatin

A549/Cis-R/BoxA_
untreated

A549/Cis-R/BoxA_
cisplatin

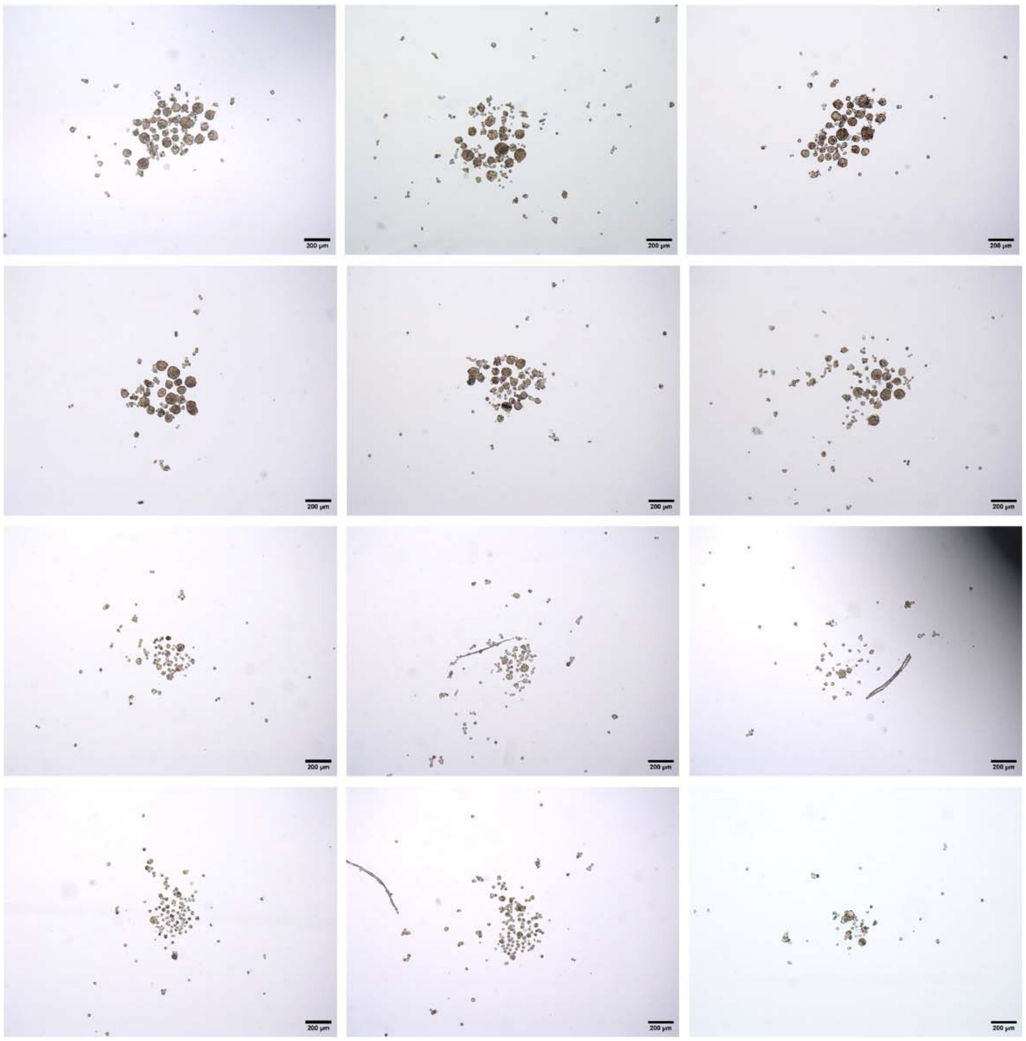

Supporting information: Representative image of sphere formation corresponding to Fig 3C

H460/Cis-R_
untreated

H460/Cis-R_
cisplatin

H460/Cis-R/BoxA_
untreated

H460/Cis-R/BoxA_
cisplatin

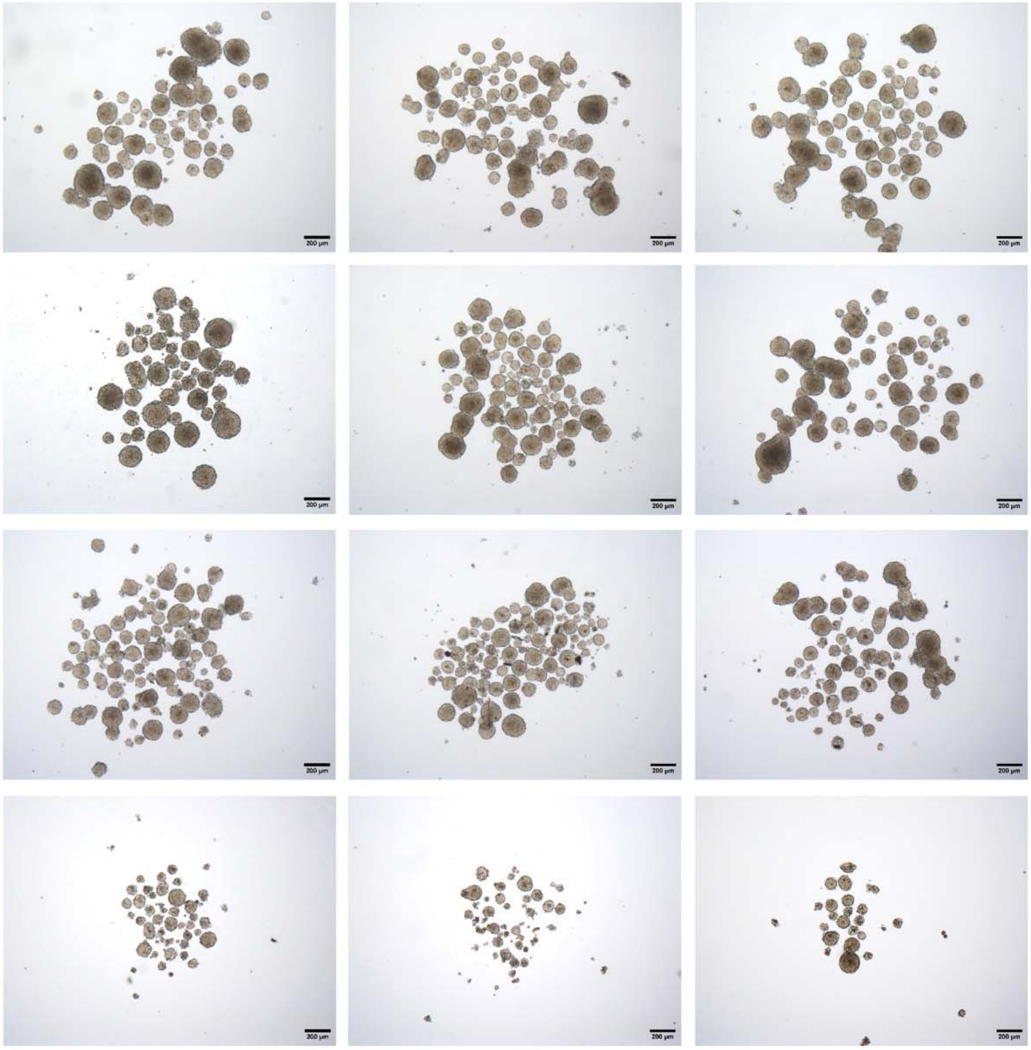

Supporting information: Representative image of sphere formation corresponding to S5A_Fig

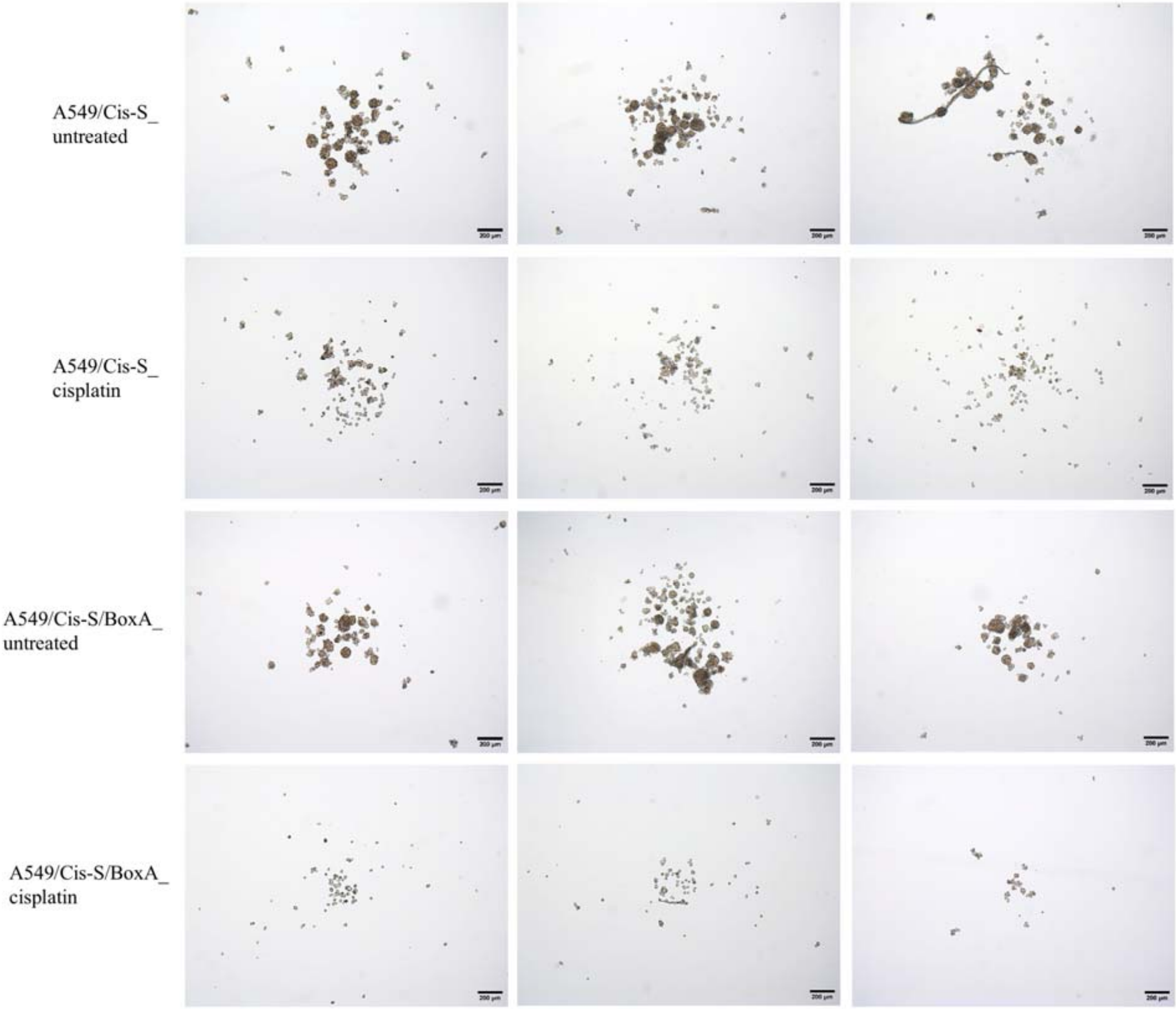

Supporting information: Representative image of sphere formation corresponding to S5C_Fig

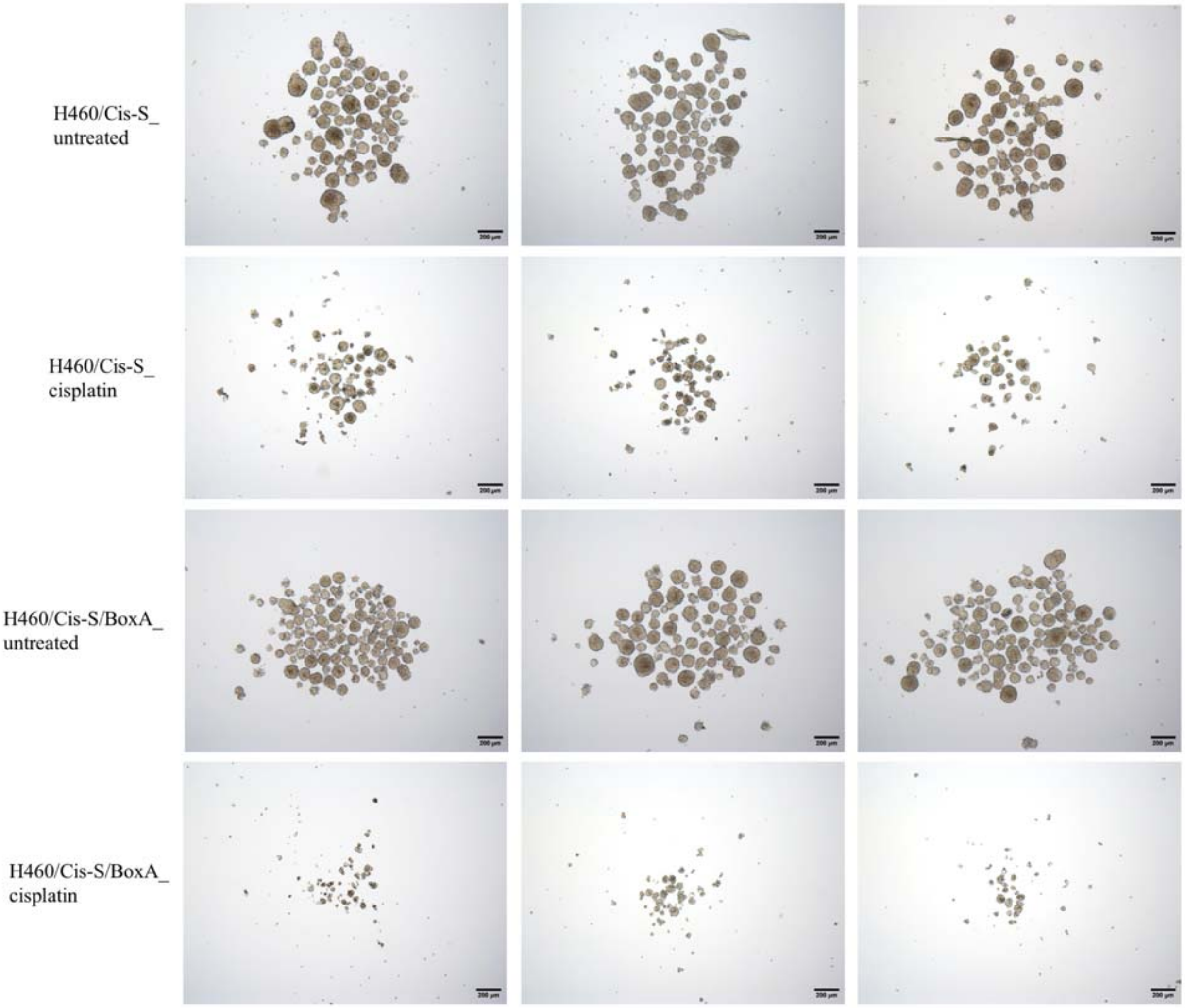

Supplement: S1 S_raw_image — (PDF) [file pone.0327144.s006.pdf]
