# Supplementary material for: HMGB1-BoxA gene therapy in reversing cisplatin resistance in non-small cell lung cancer
Source: PLoS One. 2025 Jun 25;20(6):e0327144. doi: 10.1371/journal.pone.0327144 (PMC12193834; doi:10.1371/journal.pone.0327144)
Supplement: Attachment — Submitted filename: ResponseEditor_090525.pdf [file pone.0327144.s010.pdf]

The author would like to thank the editor and reviewer for the minor revision and their suggestions. Here is the point-by-point response to editor/reviewer comments.

Response to the editor

1. We note that your Data Availability Statement is currently as follows: [All relevant data are within the manuscript and its Supporting Information files.]

Please confirm at this time whether or not your submission contains all raw data required to replicate the results of your study. Authors must share the "minimal data set" for their submission. PLOS defines the minimal data set to consist of the data required to replicate all study findings reported in the article, as well as related metadata and methods (https://journals.plos.org/plosone/s/data-availability#loc-minimal-data-set-definition).

For example, authors should submit the following data:
- The values behind the means, standard deviations and other measures reported;
- The values used to build graphs;
- The points extracted from images for analysis.

Ans: We update all the supportive information files, including raw images of the manuscript.
