# Supplementary material for: HMGB1-BoxA gene therapy in reversing cisplatin resistance in non-small cell lung cancer
Source: PLoS One. 2025 Jun 25;20(6):e0327144. doi: 10.1371/journal.pone.0327144 (PMC12193834; doi:10.1371/journal.pone.0327144)
Supplement: Attachment — Submitted filename: Review Comments to the Author (PONE-D-25-14742).docx [file pone.0327144.s008.docx]

### ****Review Comments to the Author****

The manuscript titled **“HMGB1-BoxA gene therapy in reversing cisplatin resistance in non-small cell lung cancer”**presents compelling findings on the modulation of HMGB1 localization and its role in overcoming acquired cisplatin resistance in NSCLC models. The work is timely and adds valuable insights to the understanding of chemotherapy resistance mechanisms. The manuscript is generally well-organized, and the experimental data are sound. However, I recommend the following **minor revisions** to improve clarity, data interpretation, and completeness.

#### ****1. Data Presentation and Figure Clarity****

- **Lines 169–193 (Figure 2):** Please clarify the differential impact of BoxA treatment on Cis-S vs. Cis-R cell lines. Consider adding annotations or group labels to the figure to facilitate direct comparison.
- **Lines 203–206 (Figure 3):** Include scale bars and indicate the magnification used in the tumor spheroid images to enhance reproducibility and visual interpretation.

#### ****2. Discussion – Mechanistic Clarification and Language Precision****

- **Lines 233–245:** Expand the explanation on how nuclear HMGB1 contributes to increased chemotherapy sensitivity. You may consider referencing mechanisms such as chromatin stabilization, transcriptional regulation, or modulation of DNA repair processes.
- **Lines 243–244:** The suggested mechanism involving ‘heteroduplex formation’ is interesting but speculative. Please explicitly state this as a hypothesis and recommend that future experiments validate this proposed mechanism.

#### ****3. BoxA Toxicity in Non-Cancerous Cells****

- The manuscript does not include data on the potential off-target or toxic effects of BoxA in non-cancerous cells. This is particularly relevant given its potential translational use. If such data are unavailable, consider including this as a limitation in the Discussion and propose its evaluation in future studies.
- Specifically, suggest whether experiments on normal lung epithelial cells (e.g., BEAS-2B) could help assess selective toxicity.

#### ****4. In Vivo Validation and Translational Relevance****

- While the current in vitro findings are promising, the manuscript lacks in vivo validation of BoxA’s therapeutic effect. Including (or proposing) **in vivo efficacy studies**—for instance, using cisplatin-resistant NSCLC xenograft or PDX models—would greatly strengthen the translational relevance of the work. Such studies could assess BoxA’s ability to restore cisplatin sensitivity, evaluate toxicity in normal tissues, and confirm the modulation of HMGB1 localization in a physiologically relevant context.
- If in vivo data are currently unavailable, we recommend discussing this limitation clearly and outlining future directions that involve **animal models and pharmacokinetic evaluations** of BoxA gene therapy.

#### ****5. Language, Grammar, and Abbreviation Consistency****

- **Line 34 (Abstract):** “These BoxA can potentially...” → revise to: “BoxA can potentially...”
- **Line 70:** Consider replacing “alters cisplatin resistance status” with “modifies cisplatin resistance status” for precision.
- **Lines 237–238:** The phrase “action of the molecular scissors is unlikely to be the mechanism...” can be reworded for clarity. Suggested revision:
  “The molecular scissors mechanism is unlikely to underlie the observed reversal of cisplatin resistance.”
- Ensure consistent use of abbreviations, especially Cis-S (cisplatin-sensitive) and Cis-R (cisplatin-resistant), throughout the text and figures.

### ****Overall Recommendation: Minor Revision****

The manuscript offers strong potential for advancing therapeutic strategies in cisplatin-resistant NSCLC. Addressing the points above, particularly the addition of BoxA toxicity profiling, and planning or discussing **in vivo validation**, will significantly enhance the manuscript’s impact and translational value.
